# Osteogenic Potential of Autologous Dentin Graft Compared with Bovine Xenograft Mixed with Autologous Bone in the Esthetic Zone: Radiographic, Histologic and Immunohistochemical Evaluation

**DOI:** 10.3390/ijms24076440

**Published:** 2023-03-29

**Authors:** Matko Oguić, Marija Čandrlić, Matej Tomas, Bruno Vidaković, Marko Blašković, Ana Terezija Jerbić Radetić, Sanja Zoričić Cvek, Davor Kuiš, Olga Cvijanović Peloza

**Affiliations:** 1 Doctoral School of Biomedicine and Health, Faculty of Medicine, University of Rijeka, 51 000 Rijeka, Croatia; 2 Dental Clinic Rident, 51 000 Rijeka, Croatia; 3 Department of Dental Medicine, Faculty of Dental Medicine and Health Osijek, J.J. Strossmayer University of Osijek, 31 000 Osijek, Croatia; 4 Private Practice, 51 000 Rijeka, Croatia; 5 Department of Oral Surgery, Faculty of Dental Medicine Rijeka, University of Rijeka, 51 000 Rijeka, Croatia; 6 Department of Anatomy, Faculty of Medicine, University of Rijeka, 51 000 Rijeka, Croatia; 7 Department of Periodontology, Faculty of Dental Medicine Rijeka, University of Rijeka, 51 000 Rijeka, Croatia; 8 Clinical Hospital Center Rijeka, 51 000 Rijeka, Croatia

**Keywords:** guided bone regeneration, alveolar ridge preservation, autologous dentin, bovine xenograft, CBCT, histology, BMP-4, TNF-α

## Abstract

This prospective, randomized, controlled clinical trial reports clinical, radiographic, histologic and immunohistochemical results of autologous dentin graft (ADG) and its comparison with a mixture of bovine xenograft with autologous bone (BX+AB). After tooth extraction in the esthetic zone of maxilla, the alveolar ridge of 20 patients in the test group was augmented with ADG, while 17 patients in the control group received the combination of BX+AB. Cone beam computed tomography (CBCT) was performed before tooth extraction and after 4 months when a total of 22 bone biopsies were harvested and subjected to histological and immunohistochemical analysis. Radiological analysis showed comparable results of bone dimension loss in both groups. Quantitative histologic analysis showed comparable results with no statistically significant differences between the groups. Immunohistochemical staining with TNF-α and BMP-4 antibodies revealed immunopositivity in both groups. A statistically significant difference between the groups was found in the intensity of TNF-α in the area of newly formed bone (*p* = 0.0003) and around remaining biomaterial particles (*p* = 0.0027), and in the intensity of BMP-4 in the area around biomaterial particles (*p* = 0.0001). Overall, ADG showed biocompatibility and achieved successful bone regeneration in the esthetic zone of the maxilla similar to BX+AB.

## 1. Introduction

Tooth extraction is one of the most common procedures in dentistry. Scientific studies have clearly shown that the loss of a tooth significantly alters the size of the alveolar ridge, which may be unfavorable for the placement of dental implants [1]. Although bone resorption after tooth extraction is a long-term process, statistically, the greatest loss occurs in the first month after tooth extraction. In the first six months after tooth extraction, horizontal alveolar bone loss ranges from 29% to 63% and vertical alveolar bone loss ranges from 11% to 22% [2]. According to a study conducted by Chappuis et al. [3], an alveolus with a buccal wall thickness of 1 mm or less loses 7.5 mm in height two months after tooth extraction, while an alveolus with a buccal wall thickness of 1 mm or more loses 1 mm in vertical dimension. All of the aforementioned dimensional changes have negative effects on the patient’s ability to receive prosthetic or implant-supported rehabilitation, including altered alveolar ridge morphology, atrophy, and complete disappearance of the alveolar ridge bone [2,3,4]. Due to the specific structure of the maxilla, where trabecular bone dominates, this procedure can be particularly challenging in the anterior region of this bone. Therefore, the maxilla is less resistant to resorption processes than the mandible, which is predominantly composed of cortical bone [5,6]. In addition, the anterior maxilla is also the esthetic zone, making soft and hard tissue reconstruction more challenging to meet the esthetic expectations of patients and professionals [7].

To prevent the loss of bone volume, a method known as alveolar ridge preservation is used [8]. Bone volume preservation is possible with various surgical procedures such as guided bone regeneration (GBR), with the goal of achieving successful bone regeneration according to the principles of osteogenesis, osteoinduction, and osteoconduction [9,10]. A variety of graft materials has been used to preserve the dimensions of the alveolar ridge, including autologous bone grafts, xenografts, allografts, and alloplast [11,12].

Compared to other bone graft substitutes, autologous bone is the only material that has osteogenic, osteoinductive, and osteoconductive effects [10,13]. Autologous bone is therefore the “gold standard” for augmentation of various alveolar bone defects [14,15]. However, the use of autologous bone is associated with a number of potential complications related to morbidity at the harvest or placement site, sometimes unpredictable and rapid resorption, and limited availability [15]. The incidence of complications has been reported in the literature to range from 0.5% to 10.5%, with a lower incidence of complications associated with intraoral autologous graft retrieval. Rapid resorption of autologous bone is an unpredictable and relatively common complication with an incidence ranging from 5 to 28% [16]. To reduce resorption, mixing autologous bone with bovine xenograft and using a GBR membrane is recommended, which can reduce autologous graft resorption by 50% [17].

Xenograft is a bone substitute material derived from a species different from that of the recipient. These grafts are primarily made from the inorganic portion of animal bone tissue using chemical and/or mechanical processes that remove the organic components of the bone and produce hydroxyapatite granules that resemble the human mineralized bone matrix. Xenografts have been reported to have osteoconductive properties, are hydrophilic, and biocompatible; however, bovine-derived graft biomaterials may inherently carry the risk of transmitting prion diseases to patients [18,19,20,21]. One of the most commonly used xenografts for alveolar ridge preservation today is cerabone^®^ (botiss biomaterials, Zossen, Germany). It is made from trabecular bovine bone under a a high temperature treatment (>1200 °C), which contributes to its safety and special physical properties. It has a granular structure with high porosity, which allows for good penetration of blood vessels and the formation and reorganization of newly formed bone tissue [22].

A relatively new graft material for bone regeneration is autologous dentin. In 1967, Yeomans and Urist demonstrated the presence of bone morphogenetic proteins (BMPs) in dentin, providing the first known evidence of the osteoinductive potential of the demineralized dentin matrix [23]. The only signaling molecules that can independently induce de novo bone formation at orthotopic and heterotopic sites are BMPs, and their presence in dentin distinguishes it from xenogeneic biomaterials that do not contain proteins [24,25]. Due to the development of technologies that facilitate the clinical application of dentin, it has been increasingly used as an augmentation material since 2008 [26]. The osteoconductive and osteoinductive properties of autologous dentin were determined in studies using an experimental animal model that demonstrated osteoconduction, integration and resorption of autologous dentin [27,28,29]. The use of autologous dentin in combination with platelet-rich fibrin was evaluated radiographically with cone beam computed tomography (CBCT) analysis [30]. A recent clinical study compared the clinical and histologic outcomes of augmentation of alveolar bone defects with autologous dentin and bovine xenograft [31]. Nevertheless, there is a knowledge gap regarding the osteogenic potential of autologous dentin, especially regarding the expression of inflammatory and osteoinductive factors in bone tissue after the application of autologous dentin in the alveolar socket. Therefore, the aim of this randomized controlled clinical trial was to investigate the clinical and radiographic efficacy, as well as the histological and immunohistochemical properties, of autologous dentin in alveolar ridge preservation and to compare it with bovine xenograft mixed with autologous bone.

## 2. Results

### 2.1. Clinical Evaluation

A total of 37 patients met the inclusion criteria for participation in the clinical trial. The demographic characteristics of the population and the distribution of extraction sites are shown in the tables below (Table 1 and Table 2). For each participant, only one extraction site in the esthetic zone (maxillary incisors, canines, and premolars) was considered.

The healing was uneventful. Only five patients in the test group and three patients in the control group reported moderate discomfort, pain, and swelling. Patients did not report exposed wounds or leakage of biomaterial into the oral cavity. All patients participated in regular check-ups and CBCT scans and transitioned to implant therapy after four months. At the four-month follow-up, the oral mucosa and underlying bone had completely healed in all patients.

### 2.2. Histological Results

**Figure 1:**
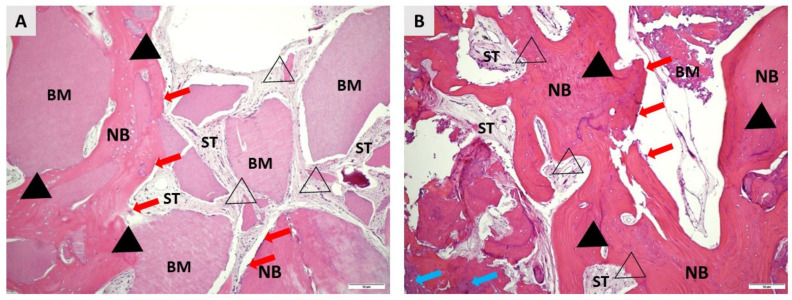
Representative photomicrographs of biopsies taken four months after alveolar ridge preservation. (**A**) shows details of the bone augmented with autologous dentin graft (ADG), whereas (**B**) shows details of bovine xenograft mixed with autologous bone. In both groups, the remaining biomaterial particles (BM and blue arrows in (**B**)) are in close contact with the newly formed bone (NB). NB has a regular, lamellar appearance in both groups and is filled with osteocytes (black fill triangles) embedded in lacunae. Osteoblasts (red arrows) are clearly visible at the margins of NB. The soft tissue areas (ST) are rich in cells, especially fibroblasts (no fill triangles). No inflammatory tissue reaction was detected in either group (hematoxylin-eosin, 100× magnification).

A total of 22 bone biopsies were obtained for histologic analysis, 12 from the test group and 10 from the control group. New bone (NB) formation was noted in both groups, extending from the apical to the coronal part of each specimen. The NB had lamellar organization and was filled with many osteocytes embedded in lacunae. Residual biomaterial (BM) was detected in close contact with NB in both groups. Many fibroblasts were present in the soft tissue (ST). No inflammatory reaction was observed in any group (Figure 1). Histomorphometric analysis revealed no statistically significant differences between the groups in the percentage of NB, BM, and ST (Table 3).

**Figure 2:**
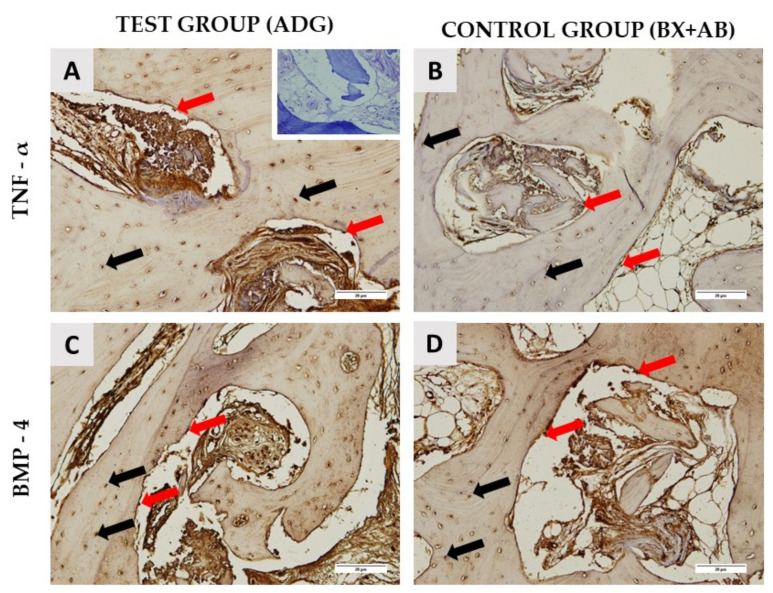
TNF-α and BMP-4 immunohistochemical staining of representative samples from the test group (**A**,**C**) and the control groups (**B**,**D**). The negative control is shown in the right upper corner of the (**A**). Red arrows indicate TNF-α- and BMP-4-positive osteoblasts located in the contact area of newly formed bone and autologous dentin graft (ADG) (**A**,**C**) or bovine xenograft mixed with autologous bone (BX+AB) (**B**,**D**). Black arrows indicate strong expression of TNF-α and BMP-4 in osteocytes embedded in the newly formed bone (**A**–**D**) (200× magnification).

Immunohistochemical analysis revealed TNF-α- and BMP-4-positive cells in the area of the new bone formation (Figure 2). Expression of TNF-α and BMP-4 was observed in both osteocytes embedded in the newly formed bone and in osteoblasts located at the bone surface. In addition, the intensity of immunohistochemical detection of TNF-α and BMP-4 was measured in the areas of newly formed bone and around the remaining biomaterial particles. A statistically significant difference in the intensity of TNF-α in the area of newly formed bone (*p* = 0.0003) and around the remaining biomaterial particles (*p* = 0.0027) and in the intensity of BMP-4 in the area around biomaterial particles (*p* = 0.0001) was observed between the test and control groups (Table 4).

### 2.3. Cone Beam Computed Tomography (CBCT) Results

A total of 37 CBCT scans were analyzed for radiographic evaluation, 20 from the test group and 17 from the control group. The measurements of alveolar ridge width before tooth extraction and 4 months after alveolar ridge preservation revealed no statistical differences in dimensional changes between the test and control groups (Table 5).

## 3. Discussion

The purpose of alveolar ridge preservation is to minimize alveolar ridge resorption after extraction [32]. Alveolar ridge preservation after tooth extraction according to guided bone regeneration (GBR) principles has been shown to be a reliable method of preventing alveolar ridge volume loss during healing [8,33]. Autologous bone is considered the gold standard among bone graft substitutes; however, due to the reported possible complications associated with its use, the focus has shifted to the development of alternatives. With this in mind, bone biopsies harvested 4 months after alveolar ridge preservation with autologous dentin graft (ADG) and bovine xenograft mixed with autologous bone (BX+AB) were examined radiographically, histologically, and immunohistochemically. To our knowledge, this is one of the first immunohistochemical reports on the use of ADG in humans, as well as the first comparison of clinical, radiographic and histologic results with the well-known and commonly used combination BX+AB. Both biomaterials were found to be effective in terms of osteoconduction and osteoinduction, and no inflammatory tissue reaction was observed. Histomorphometric and radiological results were comparable between the biomaterials, but quantitative immunohistochemical analysis examination showed statistically significant differences in TNF-α and BMP-4 expression between the analyzed biomaterials.

Depending on the preparation protocol and degree of mineralization, three types of ADG can be distinguished: demineralized dentin matrix, partially demineralized dentin matrix, and undermineralized dentin [34,35]. In this clinical study, we used a protocol that results in a demineralized dentin matrix (DDM) [36]. DDM has been shown to be superior in bone regeneration due to its irregular surface and exposed collagen fibers leading to better osteoblasts adhesion [36]. Because of the great heterogeneity of studies regarding the use of ADG and to present our results as objectively as possible, we compared them with the results of studies in which DDM was used.

The osteoinductive and osteoconductive capabilities of ADG were examined histologically in studies using an experimental animal model. ADG was found to be fully integrated into newly formed bone [29]. The studies carried out by Kim and Lee on an experimental animal model also demonstrated the ability of ADG to promote bone growth, which was related to the presence of minerals such as hydroxyapatite (HA) and tricalcium phosphate (TCP) in dentin [27,28]. In addition, studies on animal models showed that these changes began on day 14 of healing, when ADG started to be integrated into the newly formed bone [37,38]. Similar qualitative histologic observations were found in human studies [31,32]. The qualitative histologic analysis in our study revealed close contact between NB and BM, indicating successful bone regeneration and thus the osteoconductive potential of the grafts. Furthermore, no inflammatory reaction was observed in either the control or the test groups, suggesting the biocompatibility of the grafted biomaterials and the surrounding tissue. We reported that the histomorphometric result of new bone formation in the test group was 72.55 ± 12.14%. This is in accordance with findings from a recent clinical study, which exclusively examined bone tissue histomorphometrically after the use of autologous dentin, and reported 63% newly formed bone and 25% residual biomaterial 7 months after guided bone regeneration [39]. There are only few clinical studies comparing xenogeneic biomaterials with ADG. In our study, the histomorphometrical examination revealed no significant differences in the percentages of newly formed bone, residual biomaterial and soft tissue between groups. Furthermore, the results of alveolar ridge preservation with bovine xenograft BioOss^®^ and ADG were compared by Pang et al. [31], and it was found that there was no statistically significant difference in the volume of bone tissue preserved, which is in accordance with our findings.

More than 15 types of BMPs belonging to the TGF-β family have been discovered, but only three stimulate bone formation at the graft site, namely BMP-2, BMP-4, and BMP-7. Their presence stimulates mesenchymal cells to differentiate into osteoblasts, which it is strong evidence of osteoinductive properties [40]. The presence of proinflammatory cytokines such as TNF-α is essential for the initiation of bone healing, as it plays a role in the differentiation of osteoprogenitor cells into preosteoblasts [10]. Moreover, TNF-α along with RANKL plays an essential role in bone remodeling and their expression has been shown to correlate with the dynamics of bone turnover. Namely, TNF-alpha can indirectly stimulate osteoclast differentiation by itself or via RANK signaling [41,42]. In conditions of bone homeostasis, bone resorption is the most important prerequisite for bone formation. It may be that the presence of xenogeneic biomaterial in the alveolar socket caused a stronger inflammatory reaction than dentin, which was visible from the higher expression of the TNF-α immunostaining; however, it did not affect the physiology of the bone remodeling, as was evident from the achieved percentages of the newly formed bone (Table 3). Previous studies in animal models showed immunopositivity of BMP-2 and BMP-4 in the first days of bone healing after augmentation with DDM, supporting evidence of osteoinduction [29]. The immunohistochemical examination in our study revealed TNF-α and BMP-4 immunopositivity in the area surrounding biomaterial particles and the area of the newly formed bone 4 months after alveolar ridge preservation in both groups, which was confirmed by the quantitative results. These results demonstrate the osteoinductive potential in both groups. The immunopositivity in the ADG group is of additional significance because the dentin was treated with ethylenediaminetetraacetic acid (EDTA), proving that dentin can still maintain its osteoinductivity after that procedure. Interestingly, TNF-α immunopositivity around biomaterial particles and in the area of the newly formed bone was significantly higher in the BX+AB group compared to the ADG group (TNF-α ADG vs. BX+AB around BM *p* = 0.0027; TNF-α ADG vs. BX+AB around NB *p* = 0.0003) and BMP-4 immunopositivity around the biomaterial particles was significantly higher in the BX+AB group (BMP-4 ADG vs. BX+AB around BM *p* = 0.0001). This finding may be related to the presence of a bovine xenograft in the BX+AB group, which may elicit a stronger immune response, that is a common reaction to all types of substitutes and it is essential for bone healing [43].

Radiographic studies of ADG performance in the alveolar ridge are characterized by very high heterogeneity. The study by Li et al. [44] showed comparable results of marginal bone resorption at 6 and 18 months after alveolar ridge preservation using ADG and BX Pohl et al. [30] investigated dimensional changes in the alveolar ridge after socket preservation with ADG and platelet-rich fibrin (PRF). The study found that the mean changes in alveolar ridge width of 1 mm, 3 mm, and 5 mm below the crest were −1.38 ± 1.24 mm, −0.82 ± 1.13 mm, and −0.43 ± 0.89 mm, respectively. We measured the alveolar ridge at the most prominent bone points buccally and orally; therefore, an objective comparison between the aforementioned studies and our study was not possible.

From a clinical point of view, ADG shows some additional advantages compared to BX+AB. The surgical procedure (chair time) is shorter since the preparation of ADG is performed by the dental assistant during the surgery. There is also no need for additional autologous bone harvesting, which prolongs the procedure and may cause more morbidity and postoperative complications for the patient. Finally, the cost of the procedure is lower since there is no extra material used other than autologous dentin from the patient.

It should be noted that the sample size of the analyzed CBCTs and bone biopsies is small. However, we only studied the anterior part of the maxilla, which is also known as the esthetic zone and includes exclusively maxillary incisors, canines and premolars; thus, a smaller sample size is justified. In addition, this is one of the first studies to investigate TNF-α and BMP-4 expression in human alveolar bone, making this study an important demonstration of the osteogenic potential of ADG.

In conclusion, according to this study, ADG is biocompatible and has been shown to have osteoconductive and osteoinductive properties comparable to those of commonly used BX+AB (in radiological and histological comparison). The significantly higher expression of BMP-4 in the BX+AB group indicates good osteoinductive properties, due to the addition of the autologous bone. The significantly higher expression of TNF-α in the same group indicates an active bone remodeling, which has resulted in a high percentage of newly formed bone. This statistically significant difference should be further investigated and be the focus of future clinical studies.

## 4. Materials and Methods

### 4.1. Selection Criteria and Preoperative Assessment

The study was conducted in accordance with the Declaration of Helsinki. The Ethics Committee of the Faculty of Medicine of the University of Rijeka (Class: 003-08/21-01/20, No. 2170-24-04-3-21-6) and the Clinical Hospital Centre Rijeka (Class: 003-05/20-1/151, No. 2170-29-02/1-20-2) approved the conduct of the study on human participants willing to sign inform consent.

**Figure 3:**
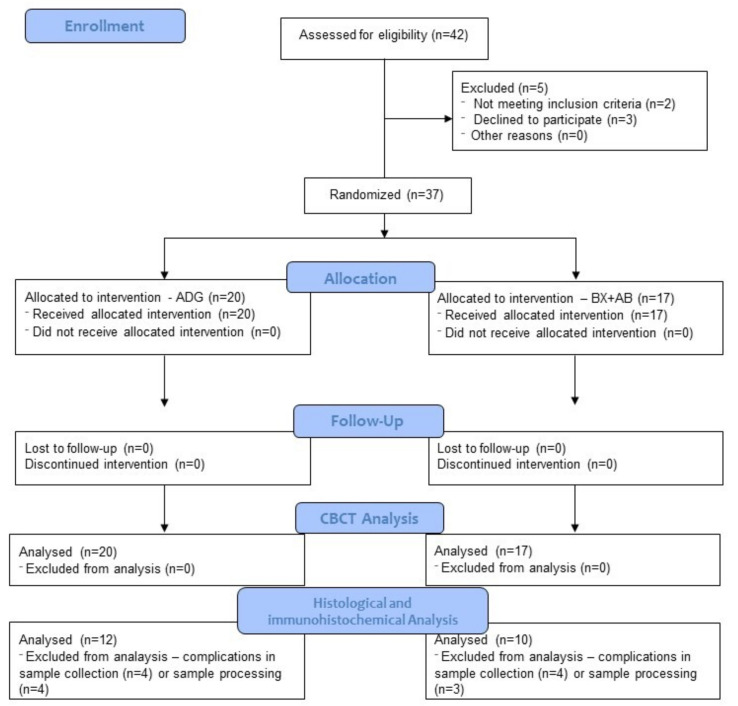
CONSORT flowchart.

Participants were consecutive individuals referred for extraction of at least one hopeless tooth in anterior maxilla followed by implant treatment. A total of 42 patients underwent the screening process; finally, 37 patients met the inclusion criteria and provided written consent to participate in clinical trial (Figure 3).

All participants were older than 18 years, healthy, and had no health problems that would exclude them from dental implants. Smokers (>10 cigarettes per day), patients with uncontrolled systemic diseases, patients who had received therapeutic radiation to the head or neck, and pregnant and lactating patients were excluded from the study. They were all treated by the same surgeon (M. O.) at the dental clinic in Rijeka, Croatia, between June 2020 and March 2022.

Before the procedure, patients underwent periodontal treatment to remove plaque and calculus and received detailed oral hygiene instructions. One hour before the procedure, patients received an appropriate antibiotic (875 mg penicillin + 125 mg clavulanic acid or 600 mg clindamycin in case of allergy). Preoperatively, 0.2% clorhexidine was used for mouth rinsing.

### 4.2. Surgical Procedure and Postoperative Follow-Up

**Figure 4:**
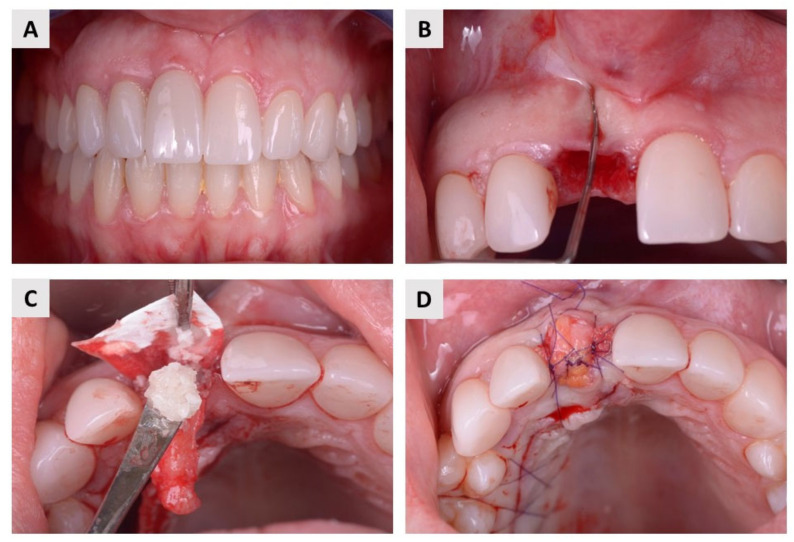
Surgical protocol in the test group. (**A**) The patient suffered from internal root resorption of the maxillary central incisor. (**B**) After atraumatic extraction, the integrity of the alveolar bone walls was checked with a dental probe. (**C**) An autologous dentin graft was placed in the extraction socket and covered with a resorbable membrane. (**D**) Finally, the wound was primary closed with connective tissue graft.

**Figure 5:**
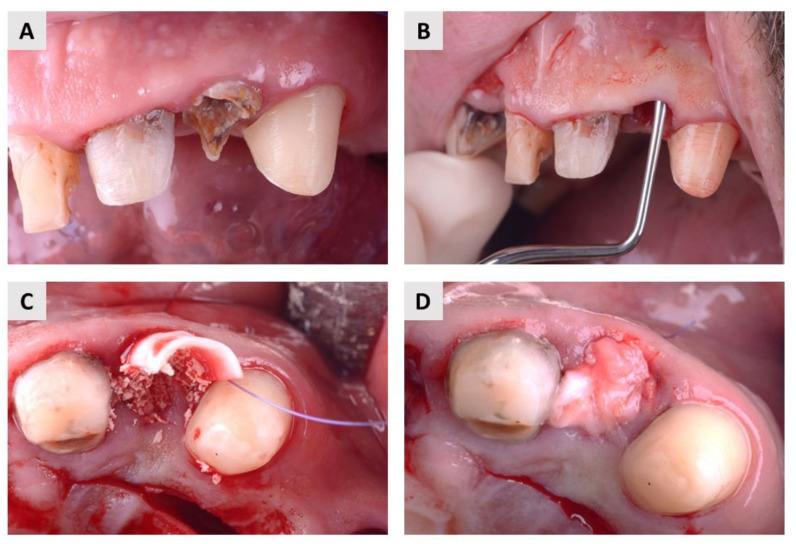
Surgical protocol in the control group. (**A**) A hopeless maxillary lateral incisor. (**B**) After extraction, a detailed curettage was carried out and the integrity of the alveolar bone walls was checked. (**C**) Bovine xenograft mixed with autologous bone and placed into extraction socket. A resorbable membrane was placed buccally. (**D**) Primary wound closure with connective tissue graft.

The local anesthetic Ubistesin forte (3M, Neuss, Germany) was used to anesthetize the area around the teeth. After a painless and atraumatic tooth extraction, the inflammatory material in the alveolus was curetted and a probe (15 UNC Colour-Coded, Hu-Friedy, Chicago, IL, USA) was used to check the integrity of all bone walls of the alveolus. Only alveoli with three intact bone walls were included in this study. After extraction and curettage, a full-thickness soft tissue flap was elevated with Curette Lucas 2.5 mm (Helmut Zepf, Seit-ingen-Oberflacht, Germany), and a resorbable collagen membrane (Biogide^®^, Geistlich Pharma, Wolhusen, Switzerland) was placed under the buccal flap. Patients were then randomized into two groups using a free randomization tool available at https://www.randomizer.org/ (accessed on 30 July 2022). Finally, in the test group, patients received an autologous dentin graft, and in the control group, they received bovine xenograft (cerabone^®^, botiss biomaterials, Zossen, Germany) mixed with autologous bone that was harvested locally, around the surgical site. In both groups, the wound was primarily closed with a resorbable collagen membrane and connective tissue graft harvested from the palate using resorbable 6.0 monofilament sutures (SMI, St. Vith, Belgium, Surgicryl Rapid 6.0.) (Figure 4 and Figure 5).

In the cases where autologous dentin graft was used, the material was prepared according to the manufacturer’s recommendations [36]. The extracted teeth were thoroughly manually cleaned with a high-speed carbide bur. Before grinding, all filling materials, calculus, caries, periodontal ligament, discolored dentin and part of the enamel were removed. After drying with an air syringe, the clean teeth were ground in a sterile chamber of the Smart Dentin Grinder (SDG) (KometaBio Inc., Cresskill, NJ, USA). The SDG device was programmed to collect 300–1200 μm particles in the collection chamber. To remove all organic debris and bacteria, the particle teeth were immersed in an alcohol-based basic cleaner for 5 min in a sterile container. They were then dried with sterile gauze. The particles were then rinsed twice in sterile phosphate-buffered saline and were ready to be used as graft material.

All patients were instructed to take the prescribed antibiotics and analgesics as needed and to rinse the mouth with chlorhexidine-based fluids for one month after treatment. Follow-up examinations were performed 10, 30, and 60 days after the procedure. Four months later, patients were called again for restoration with a dental implant.

### 4.3. Bone Biopsy Collection

**Figure 6:**
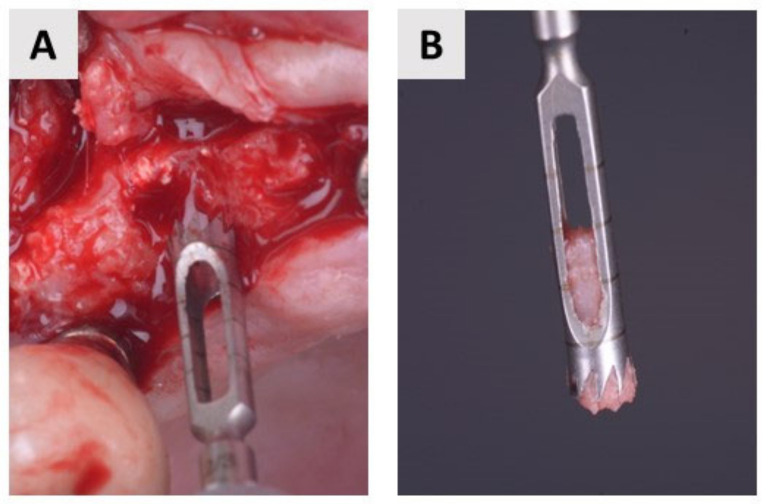
Biopsy collection. (**A**) Trephine bur in maxilla. (**B**) Closer view of the bone sample.

Bone biopsy was performed 4 months after bone augmentation simultaneously with implant bed preparation. Bone samples were harvested using a trephine burr with a diameter ranging from 2.3 to 2.8 mm (Helmut Zepf, Seitingen-Oberflacht, Germany) (Figure 6). The diameter of the trephine bur is smaller than that of the final burr in the implant set, which is important to avoid additional bone removal. The collected biopsies were preserved in 4% buffered paraformaldehyde and then forwarded to the laboratory for histological and immunohistochemical analysis. Finally, a dental implant (BEGO Semados RSX^Pro^, Bego GmbH, Bremen, Germany) was placed.

### 4.4. Histology and Immunohistochemistry

Fixed bone tissue samples were decalcified in Osteofast 2 solution (Biognost, Zagreb, Croatia), embedded in paraffin wax and cut into 5 μm thick tissue sections. Tissue sections were then stained with hematoxylin-eosin and examined under a light microscope with a video camera. For histomorphometric analysis, photomicrographs at 10× magnification were transferred to ImageJ v1.53 software (NIH, https://imagej.nih.gov/ij/). Histomorphometric analysis determined the percentage of newly formed bone, residual biomaterial, and soft tissue.

For immunohistochemical analysis, previously prepared 5 μm thick tissue sections were deparaffinized, dehydrated, and then treated with 0.3% H_2_O_2_ and incubated in citrate buffer for antigen retrieval. Sections were treated with rabbit polyclonal anti-TNF-α antibody (ab6671, Abcam, Cambridge, UK), and rabbit polyclonal anti- BMP-4 antibody (ab124715, Abcam, Cambridge, UK). After washing, incubation, and visualization with 3,3′-Diaminobenzidine (ab64238, Abcam, Cambridge, UK), the samples were embedded with entellan (Sigma-Aldrich, St. Louis, MO, USA) and examined under the light microscope in conjunction with a video camera.

Quantification of immunohistochemical staining was analyzed on previously recorded photomicrographs using the computer program cellSense v3.2. Photomicrographs taken at 200× magnification were subjected to intensity separation, its conversion to black and white, and background signal subtraction. The regions of interest were determined and placed on the obtained image display, and the final result was shown as the mean value of the color intensity—mean gray value. Histological and immunohistochemical examination were blindly performed by one investigator (O.C.P.).

### 4.5. Radiological Assessment

**Figure 7:**
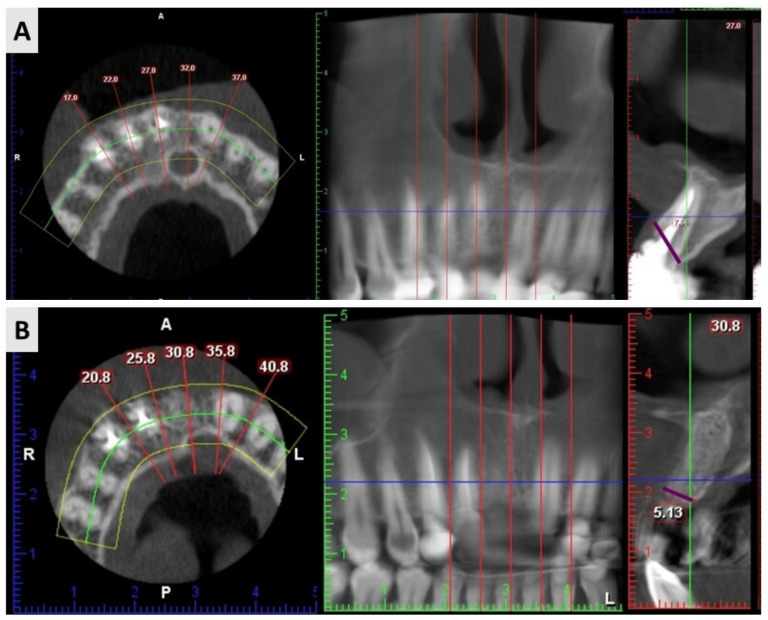
CBCT assessment protocol. The buccolingual dimension was measured between the most prominent points buccally and orally (purple marked line) at two time points—before extraction (**A**) and four months after (**B**).

Cone beam computed tomography (CBCT) scans were performed with the three-dimensional (3D) Promax (Planmeca OY, Helsinki, Finland). The scan was performed with a resolution of 0.3 mm (scan time: 8.5 s, exposure time: 4 s, 120 kV, 5 mA). Radiographic assessment was performed at two time points. The first time point was before tooth extraction, and the second was at follow-up 4 months after extraction and augmentation. Identical configurations were used in both time periods. Radiological assessment was performed by measuring the width of the alveolar ridge (buccolingual dimension) at two time points. The width of the alveolar ridge was defined as the distance between the most prominent points buccally and orally (Figure 7). All measurements were performed by a single investigator (M.O.).

### 4.6. Statistical Analysis

All data were transferred to a Microsoft Excel spreadsheet. Statistical analysis was performed using IBM SPSS Statistics (25.0, SPSS Inc., Chicago, IL, USA). Kolmogorov–Smirnov test was used to test the normality of the distribution. The data were normally distributed. The mean and standard deviation were used to present quantitative data. To compare results between groups, the Student’s *t*-test and two-tailed *t*-test were used. *p* values < 0.05 are considered significant.

## Figures and Tables

**Table 1:** Participants’ demographic information.

	ADG ^1^	BX+AB ^2^
Gender		
Female	7 (35%)	12 (70.5%)
Male	13 (65%)	5 (29.5%)
*n*	20	17
Age (years)		
Mean	52.4	55.9
SD	10.8	8.8
Min	26	38
Max	71	74

^1^ Autologous dentin graft, ^2^ bovine xenograft + autologous bone.

**Table 2:** Distribution of maxillary extraction sites.

	Incisor	Canine	Premolar	Total
ADG ^1^	12	3	5	20
BX+AB ^2^	13	3	1	17
Total	25	6	6	37

^1^ Autologous dentin graft, ^2^ bovine xenograft + autologous bone.

**Table 3:** Histomorphometrical results.

	Newly Formed Bone (NB)	Residual Biomaterial (BM)	Soft Tissue (ST)
ADG ^1^	72.55 ± 12.14%	10.61 ± 5.37%	16.84 ± 9.18%
BX+AB ^2^	69.61 ± 13.53%	12.31 ± 7.83%	18.07 ± 6.93%
*p*-value *	0.613	0.570	0.742

^1^ Autologous dentin graft, ^2^ bovine xenograft + autologous bone; * two-tailed *t* Test.

**Table 4:** Immunohistochemical intensity score results.

	Mean Value ADG ^1^ ± SD ^2^	Mean Value BX+AB ^3^ ± SD	*p* Value *
TNF-α around biomaterial	140.89 ± 4.92	149.96 ± 6.64	0.0027
TNF-α new bone area	77.49 ± 3.61	88.72 ± 7.1	0.0003
BMP-4 around biomaterial	131.26 ± 9.1	171.75 ± 10.5	0.0001
BMP-4 new bone area	108.76 ± 7.54	108.66 ± 8.2	0.9761

^1^ Autologous dentin graft, ^2^ standard deviation, ^3^ bovine xenograft + autologous bone; * Student’s *t* test.

**Table 5:** CBCT analysis of alveolar ridge dimensional changes.

	Width before Extraction	Width after Extraction	Net Change
ADG ^1^	8.06 ± 1.34 mm	7.18 ± 1.48 mm	−0.88 ± 0.76
BX+AB ^2^	7.88 ± 1.08 mm	6.64 ± 0.85 mm	−1.24 ± 0.99
*p*-value *	0.654	0.172	0.219

^1^ Autologous dentin graft, ^2^ bovine xenograft + autologous bone; * two-tailed *t* test.

## Data Availability

The data presented in this article are available on request from the corresponding author.

## References

[1] Horowitz R., Holtzclaw D., Rosen P.S. (2012). A review on alveolar ridge preservation following tooth extraction. J. Evid. Based Dent. Pract..

[2] Tan W.L., Wong T.L.T., Wong M.C.M., Lang N.P. (2012). A systematic review of post-extractional alveolar hard and soft tissue dimensional changes in humans. Clin. Oral Implant. Res..

[3] Chappuis V., Engel O., Reyes M., Shahim K., Nolte L.P., Buser D. (2013). Ridge alterations post-extraction in the esthetic zone: A 3D analysis with CBCT. J. Dent. Res..

[4] Bassir S., Alhareky M., Wangsrimongkol B., Jia Y., Karimbux N. (2018). Systematic Review and Meta-Analysis of Hard Tissue Outcomes of Alveolar Ridge Preservation. Int. J. Oral Maxillofac. Implant..

[5] Deguchi T., Takano-yamamoto T., Yabuuchi T., Ando R., Roberts W.E. (2008). Histomorphometric evaluation of alveolar bone turnover between the maxilla and the mandible during experimental tooth movement in dogs. Am. J. Orthod. Dentofac. Orthop..

[6] Nkenke E., Hahn M., Weinzierl K., Radespiel-Tröger M., Neukam F.W., Engelke K. (2003). Implant stability and histomorphometry: A correlation study in human cadavers using stepped cylinder implants. Clin. Oral Implant. Res..

[7] Cho H.L., Lee J.K., Um H.S., Chang B.S. (2010). Esthetic evaluation of maxillary single-tooth implants in the esthetic zone. J. Periodontal Implant. Sci..

[8] Kalsi A.S., Kalsi J.S., Bassi S. (2019). Alveolar ridge preservation: Why, when and how. Br. Dent. J..

[9] Darby I.B., Chen S.T., Buser D. (2009). Ridge preservation for implant therapy. Int. J. Oral Maxillofac. Implant..

[10] Dimitriou R., Tsiridis E., Giannoudis P.V. (2005). Current concepts of molecular aspects of bone healing. Injury.

[11] Haugen H.J., Lyngstadaas S.P., Rossi F., Perale G. (2019). Bone grafts: Which is the ideal biomaterial?. J. Clin. Periodontol..

[12] Yamada M., Egusa H. (2018). Current bone substitutes for implant dentistry. J. Prosthodont. Res..

[13] Shamsoddin E., Houshmand B., Golabgiran M. (2019). Biomaterial selection for bone augmentation in implant dentistry: A systematic review. J. Adv. Pharm. Technol. Res..

[14] Misch C.M. (2010). Implant Dentistry.

[15] Sakkas A., Wilde F., Heufelder M., Winter K., Schramm A. (2017). Autogenous bone grafts in oral implantology—Is it still a “gold standard”? A consecutive review of 279 patients with 456 clinical procedures. Int. J. Implant. Dent..

[16] Gultekin B.A., Bedeloglu E., Kose T.E., Mijiritsky E. (2016). Comparison of Bone Resorption Rates after Intraoral Block Bone and Guided Bone Regeneration Augmentation for the Reconstruction of Horizontally Deficient Maxillary Alveolar Ridges. Biomed. Res. Int..

[17] Maiorana C., Beretta M., Salina S., Santoro F. (2005). Reduction of autogenous bone graft resorption by means of bio-oss coverage: A prospective study-PubMed. Int. J. Periodontics Restor. Dent..

[18] Kacarevic Z.P., Kavehei F., Houshmand A., Franke J., Smeets R., Rimashevskiy D., Wenisch S., Schnettler R., Jung O., Barbeck M. (2018). Purification processes of xenogeneic bone substitutes and their impact on tissue reactions and regeneration. Int. J. Artif. Organs.

[19] Schmitt C.M., Doering H., Schmidt T., Lutz R., Neukam F.W., Schlegel K.A. (2013). Histological results after maxillary sinus augmentation with Straumann® BoneCeramic, Bio-Oss®, Puros®, and autologous bone: A randomized controlled clinical trial. Clin. Oral Implant. Res..

[20] Torres J., Tamimi F., Alkhraisat M.H., Manchón Á., Linares R., Prados-Frutos J.C., Hernández G., López Cabarcos E. (2010). Platelet-rich plasma may prevent titanium-mesh exposure in alveolar ridge augmentation with anorganic bovine bone. J. Clin. Periodontol..

[21] Lindhe J., Cecchinato D., Donati M., Tomasi C., Liljenberg B. (2014). Ridge preservation with the use of deproteinized bovine bone mineral. Clin. Oral Implant. Res..

[22] Berberi A., Samarani A., Nader N., Noujeim Z., Dagher M., Kanj W., Mearawi R., Salemeh Z., Badran B. (2014). Physicochemical characteristics of bone substitutes used in oral surgery in comparison to autogenous bone. Biomed. Res. Int..

[23] Urist M.R., SIlverman B.F., Buring K., Dubuc F.L., Rosenberg J.M. (1967). The bone induction principle. Clin. Orthop. Relat. Res..

[24] Bessho K., Tanaka N., Matsumoto J., Tagawa T., Murata M. (1991). Human dentin-matrix-derived bone morphogenetic protein. J. Dent. Res..

[25] Xiao Y.T., Xiang L.X., Shao J.Z. (2007). Bone morphogenetic protein. Biochem. Biophys. Res. Commun..

[26] Originalni R., Xhaferi B., Petreska M.P., Xheladini A., Papić I. (2021). Use of mineralized dentin graft in augmentation of different indication areas in the jaw bones. Serb. Dent. J..

[27] Kim J.-Y., Kim K.-W., Um I.-W., Kim Y.-K., Lee J.-K. (2012). Bone Healing Capacity of Demineralized Dentin Matrix Materials in a Mini-pig Cranium Defect. J. Korean Dent. Sci..

[28] Lee D.H., Yang K.Y., Lee J.K. (2013). Porcine study on the efficacy of autogenous tooth bone in the maxillary sinus. J. Korean Assoc. Oral Maxillofac. Surg..

[29] Abrantes Pinheiro Carvalho V., De Olivera Tosello D., de Castillo Salgado M.A., Fernandes Gomes M. (2004). Histomorphometric analysis of homogenous demineralized dentin matrix as osteopromotive material in rabbit mandibles. Int. J. Oral Maxillofac. Implant..

[30] Pohl S., Binderman I., Tomac J. (2020). Maintenance of Alveolar Ridge Dimensions Utilizing an Extracted Tooth Dentin Particulate Autograft and Platelet-Rich fibrin: A Retrospective Radiographic Cone-Beam Computed Tomography Study. Materials.

[31] Pang K.M., Um I.W., Kim Y.K., Woo J.M., Kim S.M., Lee J.H. (2017). Autogenous demineralized dentin matrix from extracted tooth for the augmentation of alveolar bone defect: A prospective randomized clinical trial in comparison with anorganic bovine bone. Clin. Oral Implant. Res..

[32] Esposito M., Grusovin M., Worthington H., Coulthard P., Esposito M. (2006). Interventions for replacing missing teeth: Bone augmentation techniques for dental implant treatment. Cochrane Database of Systematic Reviews.

[33] Avila-Ortiz G., Chambrone L., Vignoletti F. (2019). Effect of alveolar ridge preservation interventions following tooth extraction: A systematic review and meta-analysis. J. Clin. Periodontol..

[34] Cenicante J., Botelho J., Machado V., Mendes J.J., Mascarenhas P., Alcoforado G., Santos A. (2021). The Use of Autogenous Teeth for Alveolar Ridge Preservation: A Literature Review. Appl. Sci..

[35] Sánchez-Labrador Martínez de Morentin L., Martínez-Pereda M., Martínez L.-Q. (2019). Use of Autogenous Dentin as Graft Material in Oral Surgery Martín-Ares, María Martínez-González, José María. Cient. Dent..

[36] Binderman I., Hallel G., Nardi C., Yaffe A., Sapoznikov L. (2014). A novel procedure to process extracted teeth for immediate grafting of autogenous dentin. J. Interdiscipl. Med. Dent. Sci..

[37] De Oliveira G.S., Miziara M.N., Silva E.R.D., Ferreira E.L., Biulchi A.P.F., Alves J.B. (2013). Enhanced bone formation during healing process of tooth sockets filled with demineralized human dentine matrix. Aust. Dent. J..

[38] Reis-Filho C.R., Silva E.R., Martins A.B., Pessoa F.F., Gomes P.V.N., De Araújo M.S.C., Miziara M.N., Alves J.B. (2012). Demineralised human dentine matrix stimulates the expression of VEGF and accelerates the bone repair in tooth sockets of rats. Arch. Oral Biol..

[39] Mazor Z., Horowitz R., Prasad H., Kotsakis G. (2019). Healing Dynamics Following Alveolar Ridge Preservation with Autologous Tooth Structure. Int. J. Periodontics Restor. Dent..

[40] Derner R., Anderson A.C. (2005). The bone morphogenic protein. Clin. Podiatr. Med. Surg..

[41] Boyce B.F., Xing L. (2008). Functions of RANKL/RANK/OPG in bone modeling and remodeling. Arch. Biochem. Biophys..

[42] Algate K., Haynes D.R., Bartold P.M., Crotti T.N., Cantley M.D. (2016). The effects of tumour necrosis factor-α on bone cells involved in periodontal alveolar bone loss; osteoclasts, osteoblasts and osteocytes. J. Periodontal Res..

[43] Rodriguez A.E., Nowzari H. (2019). The long-term risks and complications of bovine-derived xenografts: A case series. J. Indian Soc. Periodontol..

[44] Li P., Zhu H.C., Huang D.H. (2018). Autogenous DDM versus Bio-Oss granules in GBR for immediate implantation in periodontal postextraction sites: A prospective clinical study. Clin. Implant. Dent. Relat. Res..

